# The Examination of Fæces

**Published:** 1907-09-28

**Authors:** 


### Points in Pathology.

### III.—THE EXAMINATION OF FÆCES (*continued from p. 535*).

As a routine practice the fæces should be examined much more regularly than is usually done. It is perhaps of less importance in the diseases of temperate climates than in those of the tropics, but still considerable information may often be obtained and obscure points cleared up by attending to this. For an ordinary macroscopic inspection the stools should be placed in large flat glass dishes with tight-fitting lids in order that one may readily see them, but still avoid the unpleasant odour. During the examination of the patient, or after this is concluded, in hospitals where fæces are regularly studied, the nurse brings round the dish and then the physician sees for himself how things are going on, and can thus also readily demonstrate to students or others the points to be noted. The most important of these are the colour, consistence, form, the presence or absence of bile, and of abnormal constituents such as blood, mucus, shreds of tissue or sloughs, parasitic worms, either complete or in parts, and so on. The only way to tell whether a case of dysentery, say, or of *sprue* is progressing favourably under treatment is daily to inspect the stools; in the former the gradual disappearance of the blood, mucus, pus, and sloughs, and the return to a solid condition, indicates that repair is taking place in the damaged bowel while in the latter the reappearance of bile and the disappearance of the diarrhœa are phenomena of equally hopeful import.

### Intestinal Parasites.

After the administration of anthelmintics for the different intestinal entozoa careful examinations of the fæces must also be made to determine the presence or absence of the head of a tape-worm, or the number of adult oxyures (thread-worms) or ankylostomata (hook-worms). The best way of doing this is to strain the stool through fairly fine muslin by repeated washings, then to place what remains behind in a flat glass dish, mixing this with water and placing it over a black glass slab or a dark-coloured table. The worms then will readily be detected by their white colour, and may be picked out with a fine pair of forceps, washed again, counted, and preserved if necessary. The demonstration of the head of the tape-worm requires some skill. One usually finds on following up the segments that the thin part of the neck bearing the head has got broken off, and then the question arises, Has this remained behind in the body or is it lying free in the fæces? If in the latter, by following out the above plan it will be found; it is about the size of an ordinary pin-head with a piece of the neck about the thickness of a piece of catgut attached: while if not here it means that it has not become detached from the intestine, and in due course a new worm will grow from it and require subsequent treatment. This sequence of events is common, so it is useful to be able definitely to tell the patient whether or not the head has been passed. Flukes are not common in the human subject in England; they are shaped like leaves. The ascaris lumbricoides or common round worm is seen fairly frequently; on account of its large size it cannot be overlooked. The trichocephalus dispar, or whip-worm, is not expelled after anthelmintics; its presence, however, may be detected by finding its eggs in the fæces (see later), or it may be picked out of the cæcum at autopsy.

### Bacteria in the Stools.

After the completion of the macroscopical examination of the fæces—the importance of which has just been indicated—we may proceed to its microscopical study, and further valuable details may thus be gained. Specimens may be prepared either wet or dry, unstained or stained, just as with blood, urine, and other excretions: for the former all that is required is to take up some of the fæces with a match or a platinum loop, put a drop of it on a slide, cover this with a slip, and ring with vaseline. If too hard or dry it may be softened by mixing with a little water. For the latter films are made on slides as with sputum, fixed by heat or alcohol, and then stained by any of the simple bacterial stains or by Leishman's modification of Romanowsky's stain. Whichever method is adopted, the first noticeable thing is the enormous numbers of bacteria that are present: the technique for the isolation and differentiation of these is difficult, tedious, and of little value unless carried out by a skilled bacteriologist. The bacilli of typhoid, dysentery, cholera, etc., may thus be recovered from the stools.

Leaving the bacteria, then, other abnormal constituents may be detected, but considerable practice is required to tell them from ordinary vegetable cells and fibres normally found in the stools. The most important are parasites of various kinds, protozoa or the ova of various helminths. Of the former we have the amœbæ, one of which, the amœba coli, is the causative agent in the tropical variety of dysentery. They are sometimes found in great abundance, at other times they are scanty and require careful looking for. To make certain that they are present it is necessary to heat the slide to blood temperature in order that they may move. For this purpose it should be laid upon a copper stage with a circular hole cut in the middle, a long arm projecting from its anterior aspect, so that this may be placed in a Bunsen burner standing somewhere near the microscope. The heat passes along this to the copper stage, and so to the glass slide, and then the parasites will begin to throw out their pseudopodia and move about among the cells and other debris. The ova of the different worms are easily detected; no heating is required, and most can be seen with the low powers of the miscroscope. The commoner of these are the ankylostoma duodenale, ascaris lumbricoides, trichocephalus dispar, oxyuris vermicularis, bilharzia hæmatobia, tænia solium, and tænia saginata. The ankylostome eggs have a thin shell, and the protoplasm inside is usually segmented into two or three masses; those of the common round worms have no segmentation of the protoplasm, are usually deep yellow in colour, and have an irregular kind of sheath round the shell, while the trichocephalus ova, the prevailing colour of which is brownish yellow, are shaped like little barrels with a knob at each end. The eggs of the bilharzia are furnished with a spine, which may in some instances be terminal, in others lateral. A careful search for such eggs should always be made in patients with rectal trouble who have resided in Egypt or other parts of Africa. The eggs of the two tapeworms mentioned above resemble each other closely; from the others they can be differentiated by their shape, which is circular to slightly oval, by the radially striated shell, and by the presence of hooklets in the interior. Occasionally living embryos are seen in the stools; they are usually the larval forms of the Rhabdonema Intestinale, and are known by the name of the Anguillula stercoralis.

